# Role of nitric oxide and related molecules in schizophrenia pathogenesis: biochemical, genetic and clinical aspects

**DOI:** 10.3389/fphys.2015.00139

**Published:** 2015-05-11

**Authors:** Regina F. Nasyrova, Dmitriy V. Ivashchenko, Mikhail V. Ivanov, Nikolay G. Neznanov

**Affiliations:** V.M. Bekhterev Saint Petersburg Psychoneurological Research InstituteSaint Petersburg, Russia

**Keywords:** schizophrenia, nitric oxide, nitric oxide synthase, oxidative stress, NO, NOS, genetics, pathogenesis

## Abstract

Currently, schizophrenia is considered a multifactorial disease. Over the past 50 years, many investigators have considered the role of toxic free radicals in the etiology of schizophrenia. This is an area of active research which is still evolving. Here, we review the recent data and current concepts on the roles of nitric oxide (NO) and related molecules in the pathogenesis of schizophrenia. NO is involved in storage, uptake and release of mediators and neurotransmitters, including glutamate, acetylcholine, noradrenaline, GABA, taurine and glycine. In addition, NO diffuses across cell membranes and activates its own extrasynaptic receptors. Further, NO is involved in peroxidation and reactive oxidative stress. Investigations reveal significant disturbances in NO levels in the brain structures (cerebellum, hypothalamus, hippocampus, striatum) and fluids of subjects with schizophrenia. Given the roles of NO in central nervous system development, these changes may result in neurodevelopmental changes associated with schizophrenia. We describe here the recent literature on NOS gene polymorphisms on schizophrenia, which all point to consistent results. We also discuss how NO may be a new target for the therapy of mental disorders. Currently there have been 2 randomized double-blind placebo-controlled trials of L-lysine as an NOS inhibitor in the CNS.

## Introduction

### The pathogenesis of schizophrenia. basic theories of development of the disease

Schizophrenia is a severe mental disease with a chronic course, mainly manifesting at a young age (Tien and Eaton, 1992). According to various data, about 0.3–0.7% of the world's population suffer from schizophrenia (McGrath et al., 2008; Tandon et al., 2008). Aetiopathogenetic mechanisms of this disease are still unclear (Eaton et al., 1995; Keshavan et al., 2013). There is also a generality of symptoms between schizophrenia proper, schizoaffective disorder, bipolar affective disorder (Rimol et al., 2010; Keshavan et al., 2013). In the present circumstances, there is a need for a more precise answer to the question of what underlies the pathogenesis of schizophrenia and defines its uniqueness.

Currently, schizophrenia is considered a multifactorial disease. Up to 80% of cases of schizophrenia are associated in one way or another with genetic factors (Sullivan et al., 2003; Käkelä et al., 2014). Recent GWAS-analyses have revealed genes associated with schizophrenia (for example, NRGN, TCF4, and TSNARE1). The functions of some genes are still unknown (Stefansson et al., 2009; Sleiman et al., 2013). Therefore, the known genetic risk cannot yet predict the cause of the disease and what underlies its pathogenesis. There are several basic hypotheses regarding the development of schizophrenia. The earliest hypothesis postulates that the cause of the disease consists of dopamine metabolism disturbance, namely in the increase in its synthesis (van Rossum, 1966; Creese et al., 1976). The second version of the same hypothesis argues that dopamine metabolism is disturbed in two directions—the level of the neuromediator increases in subcortical (striatum) and decreases in prefrontal structures of the brain (Scatton et al., 1982). In particular, the hypothesis attempted to associate negative symptoms with low-dopaminergiñ state (Davis et al., 1991). Finally, the dopaminergic theory was reduced to the concept of “a final general way” that is interpreted as an influence of various factors, including exogenous factors, on presynaptic dopamine neurotransmission in striatum and that leads to development of the disease (Howes and Kapur, 2009). It is possible to consider the recognition of the role of glutamate (NMDA-receptors) and gamma-aminobutyric acid (GABA) as an addition to the dopaminergic theory of schizophrenia in its pathogenesis (Timms et al., 2013; Hu et al., 2014). Another hypothesis is associated with detection of development disturbances of some brain areas in schizophrenics (Pino et al., 2014). The latter hypothesis is backed by a lot of evidence (Howes et al., 2011; Jenkins, 2013), especially in the aspect of neurocognitive impairment (Dickson et al., 2012). A related point of view is a neurodegenerative theory of the disease. The essence of the theory is that schizophrenia is not a consequence of the improper development, but of gradual progressive disintegration of the nervous tissue in the brain (Hardy and Gwinn-Hardy, 1998). In the latter two theories, the important role is attributed to exogenous factors (Jenkins, 2013). Taking into account available evidence, it is possible to assume that etiopathogenesis of schizophrenia involves influence of the environment on the organism both during the process of development and after its completion. Of major significance are alterations in gray matter, occurring in the critical periods of development—during childbirth, at adolescence (Clarke et al., 2011; Hart et al., 2013). But pathogenic mechanisms which lead to described disturbances are the subjects of discussions. Publications of recent years state the undoubted role of inflammation and oxidative stress in pathogenesis of mental diseases, especially schizophrenia (Song et al., 2013; Bergink et al., 2014; Haller et al., 2014). In numerous investigations, associations between existence of disease and activation of immune markers in the patient have been found (Song et al., 2013; Bergink et al., 2014; de Witte et al., 2014). Some authors consider infectious agents as etiologic factors (Brown and Derkits, 2010). But, irrespective of its cause, the inflammatory process is associated with oxidative stress which leads to alterations of the tissues, some of which are irreversible (Bitanihirwe and Woo, 2011).

### Role of oxidative stress of CNS in schizophrenia development

Role of toxic free radicals in the etiology of schizophrenia was first investigated more than 50 years ago (Hoffer et al., 1954). As of now, markers of oxidative stress are revealed in patients with schizophrenia, reliably more often than in the general population. One of the latest papers is a meta-analysis by Flatow et al. (2013), where 44 investigations are considered and a conclusion about reliability of association of markers of oxidative stress with psychosis and schizophrenia existence in patients is drawn (Flatow et al., 2013). Let us consider in more detail what is included in concept of oxidative stress and what its potential role in schizophrenia development is.

## The structure of oxidative stress

### Free radicals

Oxidative stress is a state during which the balance between the system of antioxidants and level of free radicals, able to damage tissues, is disturbed (Kohen and Nyska, 2002). Synthesis of free radicals occurs during physiological oxidation-reduction processes (Berg et al., 2004). Free radicals are as follows: active forms of oxygen (reactive oxygen species—ROS), active forms of nitrogen—(RNitrogenS—RNS), carbon-centered radicals and sulfur-centered radicals (Miller et al., 1990). ROS is the most significant system of free radicals, because it shows the double role of oxygen in the organism (oxygen paradox)—this element is involved almost in all vital processes, and at the same time its active forms, leading to damage of tissues, are formed (Davies, 1995). Hydrogen peroxide (H_2_O_2_), superoxide-radical (O2^·^−) and hydroxide-radical (OH^·^) belong to ROS. Nitrogen-containing factors of oxidation: nitrogen oxide (NO), peroxynitrite (ONOO^·^) and nitrogen dioxide (N_2_O_2_). Nitric oxide is able to form hydroxyl radicals (at the expense of the unpaired electron) and to turn into nitrogen dioxide (Wu et al., 2013). During maintenance of balance, free radicals carry out important functions in the organism: they protect cells against infectious and foreign agents, serve as secondary mediators in regulation of activity of cardiovascular system, are involved in processes such as maintenance of intracellular level of calcium, phosphorylation/dephosphorylation of proteins and activation of transcription factors (Halliwell, 2007; Valko et al., 2007; Ataya et al., 2011; Wu et al., 2013). Nitric oxide possesses properties of both pro- and antioxidant. So, it is able to stimulate peroxidation of lipids and mediate antioxidant reactions in cellular membranes at the same time (Radi et al., 1991). NO is able to bind with peroxyl radicals, interrupting the oxidative process circuit. However, free nitric oxide can bind with superoxide, forming highly toxic peroxynitrite (Bitanihirwe and Woo, 2011). The imbalance of antioxidants and free radicals leads to damage of cellular structures: proteins, lipids, DNA (Kohen and Nyska, 2002).

### Antioxidants

The antioxidants, serving for maintenance of oxidation-reduction balance, are as follows: superoxide dismutase (SOD), catalase (CAT) and glutation peroxidase (GSH-Px) (Yao and Keshavan, 2011). The three enzymes act in cooperation, blocking formation of free radicals at different stages of their metabolism. Hydrogen peroxide formed by SOD is decomposed into water and oxygen by catalase. Breakage of this mechanism of protection leads to activation of peroxidation of lipids. GSH-Px is able to stop the process at this stage with the use of the transfer of toxic hydroperoxides into less active forms. Disturbance of activity of any of three enzymes results in increased vulnerability of membranes of cells for damage by free radicals (Smith, 1992). Albumin, vitamin E (α-tocopherol), uric acid, bilirubin, ascorbic acid, and thioredoxin belong traditionally to nonenzyme antioxidants (Yao et al., 2000). Nonenzyme antioxidants from plasma of blood make the basic contribution to counteraction to oxidative stress (Yao et al., 2000; Reddy et al., 2003; Reddy, 2011).

## Oxidative stress and schizophrenia

The role of oxidative stress in the development of mental diseases is still debated despite decades of investigations in this direction. The different approaches of researchers appear to be a likely reason for these debates. Free radicals dissociate very quickly and measuring their number in live organisms is difficult. Thus, indirect methods such as measurement of activity of basic antioxidant enzymes as well as the quantitative identification of peroxidation products in biological mediums are more widespread (Wu et al., 2013). The role of oxidative stress is actively studied in the terms of neuropsychiatric diseases, such as depression (Moylan et al., 2014; Rodrigues et al., 2014), bipolar disorder (Bauer et al., 2014), Alzheimer's disease (Rodrigues et al., 2014), and Huntington's chorea (Ciancarelli et al., 2014). In this review we will go into detail on schizophrenia, without mentioning the investigations, covering other diseases.

### Indicators of activity of antioxidant enzymes in schizophrenia

As mentioned above, the measurement of antioxidant enzymes is complicated due to their properties. However, numerous studies have been carried out in this field. Unfortunately, an unequivocal answer still has not been obtained. Studies of SOD activity in patients with schizophrenia are met in literature more often than that of other enzymes. Many authors have concluded that activity of this enzyme is reduced in patients with schizophrenia (Ben Othmen et al., 2008; Dadheech et al., 2008; Raffa et al., 2009, 2012a; Gonzalez-Liencres et al., 2014; Wu et al., 2014). However, there are results for both hyperactivity of superoxide dismutase in this disease (Wu et al., 2012) and unaltered activity as compared to controls (Raffa et al., 2011; Srivastava et al., 2001). Results regarding measuring levels of glutation peroxidase (Raffa et al., 2009, 2012b; Dietrich-Muszalska and Kwiatkowska, 2014) and catalase (Ben Othmen et al., 2008; Raffa et al., 2011, 2012b) are contradictory. This is covered in more detail in relevant literature reviews (Yao and Keshavan, 2011; Wu et al., 2013). To present, 2 meta-analyses of research of activity of antioxidants in patients with schizophrenia have been carried out. Zhang et al. (2010) concluded that oxidative stress in schizophrenia really takes place. However, decrease in SOD activity in this disease has been recognized as reliable, levels of CAT and GSH-Px have not shown sufficient statistical effect (effect size). Authors indicate that considerable heterogeneity of the carried-out analysis means an influence of unaccounted factors on the obtained result (Zhang et al., 2010). Meta-analysis by Flatow et al. (2013), mentioned above, has confirmed decrease in SOD activity in patients with schizophrenia according to cross-sectional investigations in the first psychotic episode, in remission and in the state of exacerbation. But considerable additions to results of the previous systematic review have been made: activity of catalase was reliably decreased in patients with initial episode (first episode psychosis) (*p* < 0.01) and reliably increased in patients in remission (*p* < 0.01) as compared with controls. Authors have assumed that the total antioxidant level is a situationally conditioned marker, labile under the influence of the mental state of the patient. But enzyme SOD, which has shown, irrespective of disease's stage, decreased as compared with healthy controls activity level, can be considered as a typical trait of patients with schizophrenia (Flatow et al., 2013).

Peroxidation products were also studied as markers of oxidative stress. The basic objective of these investigations—malondialdehyde (MDA), identified with use of measuring of the content of thiobarbituric acid reactive substance (TBARS) (Vasankari et al., 1995). Investigations are so far not plentiful. Gubert et al. (2013) have found a reliable increase in the level of TBARS in patients with schizophrenia as compared with controls (Gubert et al., 2013). This result coincides with previously obtained results (Zhang et al., 2006; Dietrich-Muszalska and Kontek, 2010), including meta-analysis by Zhang et al. (2010). In addition, seasonal dynamics of level of metabolites in plasma of patients with schizophrenia have been followed up: in summer, contents of MDA in serum exceeds winter indices by 33,9% (Morera et al., 2009). Besides MDA, isoprenes, in particular 8-isoPGF2 alpha, were considered as biomarkers of peroxidation (Dietrich-Muszalska and Olas, 2009). However, there is still not enough of an evidence base for association of 8-isoPGF2 alpha with schizophrenia.

### Indices of activity of plasma antioxidants in schizophrenia

As mentioned above, plasma antioxidants make the basic (>85%) contribution to the fight against oxidative stress (Wayner et al., 1987; Reddy et al., 2003; Reddy, 2011). For patients with schizophrenia, it is shown that in the first psychotic episode, plasma levels of α-tocopherol (Dadheech et al., 2008), ascorbic acid decrease (Suboticanec et al., 1990; Dadheech et al., 2008), level of thioredoxin increases (Zhang et al., 2009; Owe-Larsson et al., 2011). In opinion of Zhang et al. (2009), in patients with schizophrenia, the level of thioredoxin is close to that of healthy controls (Zhang et al., 2009). The same authors, in 2013, have confirmed that the plasma level of thioredoxin in patients with schizophrenia does not exceed reliably healthy controls and is associated with the level of cognitive abilities. It is shown that the level of thioredoxin is reliably decreased in patients with attention deficit disorder (Zhang et al., 2013). Bilirubin, uric acid and albumin have also been measured in patients with schizophrenia. Decreased plasma levels in the first psychotic episode as compared with controls have been noted (Yao et al., 1998, 2000; Reddy et al., 2003; Pae et al., 2004). It is interesting to note that attempts to associate separate symptoms of schizophrenia with levels of plasma antioxidants and the use of these symptoms as a diagnostic and predictive marker have taken place (Yao et al., 2012). Indeed, the total antioxidative status (TAS) is reliably low in the first psychotic episode, negatively correlating with negative symptoms according to PANSS (Positive and Negative Syndrome Scale) (Pazvantoglu et al., 2009; Li et al., 2011). The assumption has been made that oxidative stress arises during onset of the disease and exerts essential impact on the further course of schizophrenia, especially on the formation of negative symptoms. Preliminary results of the investigation by Albayrak et al. (2013) have revealed that patients with persistent negative symptoms (deficit schizophrenia) have a decreased total antioxidative potential and, at the same time, their index of oxidative stress is increased as compared with healthy controls and patients with non-deficit schizophrenia (Albayrak et al., 2013).

Influence of oxidative stress on the course of schizophrenia is confirmed by studies which show that treatment with antipsychotics leads to considerable alterations of the oxidation-reduction balance. However, results indicate otherwise. There is a point of view that antipsychotic therapy decreases the level of oxidants in the organism (Tsai et al., 2013). At the same time, pro-oxidant activity is attributed to Haloperidol *in vitro* as compared with Quetiapine and Olanzapine (Dietrich-Muszalska, 2011). Clozapine has contradictory properties regarding influence on oxidative stress (Miljevic et al., 2010; Gilca et al., 2014). But despite all contradictions, an undoubted effect of treatment with antipsychotics on indicators of oxidative stress is confirmed.

It is clear from the above paragraphs that there is a sufficient number of markers of oxidative stress, and this is an almost unlimited field for research. However, in this review we would like to consider the system of nitric oxide and related molecules within the pathogenesis of schizophrenia in more detail.

## Nitric oxide in pathogenesis of schizophrenia

### Biochemistry of nitric oxide in the organism and CNS. the interactions of NO with other factors of oxidative stress

Before considering the works devoted to the role of nitric oxide in development of schizophrenia, it is necessary to understand its biochemistry, biological parameters and functions.

Nitric oxide is involved in many processes occurring in the brain: regulation of synaptic plasticity (Hölscher and Rose, 1992), release of mediators (Lonart et al., 1992), and development of nervous tissue (Gibbs, 2003). Below we describe the main points of NO's biochemistry and its role in CNS.

#### Biochemistry of NO

Nitric oxide (NO) is released during conversion of L- arginine into L-citrulline under influence of nitric oxide synthase (NOS) (Ghafourifar and Cadenas, 2005). The reaction proceeds in the presence of oxygen, NADPH-containing flavin adenine dinucleotide (FAD), flavin mononucleotide (FMN), heme, thiol and tetrahydrobioprotein as cofactors (Akyol et al., 2004; Giraldi-Guimarães, 2004). NO has a very short half-life period—several seconds in water medium. However, placed under anaerobic conditions, this radical does not decompose for more than 15 s. In mixed water and lipid medium, nitric oxide is easily able to penetrate cytoplasmic membranes (Chiueh, 1999; Valko et al., 2007; Babicová et al., 2013). Free radical NO at the expense of unpaired electron possesses toxic potential. So, in high concentrations, nitric oxide reacts with superoxide-radical, forming peroxynitrite (ONOO) (Ridnour et al., 2004; Blaise et al., 2005; Wu et al., 2013), a powerful oxidant at physiological pH (Noack et al., 1999). Peroxynitrite is able to trigger lipid peroxidation and destruction of proteins, amino acids, nucleic acids; this leads to suppression of activity of enzymes and autoxidation of dopamine. This mechanism has been studied in the context of development of neurodegenerative diseases, in particular, Parkinson's disease (Tohgi et al., 1998). However, peroxynitrite quickly turns into stable nitrate NO_3_- (Tohgi et al., 1998; Antunes et al., 2005). Toxicity of NO increases during low concentrations of L- arginine, as in this case superoxide-radical is generated by NOS (Xia et al., 1998; Akyol et al., 2004). The state in which formation of reactive compounds of nitrogen (RNS) exceeds their neutralization is called nitrosative stress (Ridnour et al., 2004; Valko et al., 2007). It is worth mentioning that most physiological effects of NO are mediated by a radical that quickly binds with complexes: Fe^2+^-Haem. The reaction product is {Fe2+-NO^·^}. This compound allows avoiding reactions of oxidative stress (Archer, 1993; Valko et al., 2007).

#### NO-synthase

The enzyme generating nitric oxide is represented in the organism in the kind of three isoforms: neuronal (nNOS), inducible (iNOS), and endothelial (eNOS).

Nitric oxide synthesized by endothelial NO synthase renders vasodilatating, anti-inflammatory, antithrombotic and antiproliferative effects. Endothelial NO synthase itself is located in the cells of endothelium (Furchgott and Zawadzki, 1980; Endres et al., 2004). Synthesis of nitric oxide by endothelial NOS is regulated by several factors: calcium-dependent calmodulin, caveolin-1 and 3, B2-receptors of bradykinin, A1-receptors of angiotensin, steroid hormones (Fleming and Busse, 2003; Gwathmey et al., 2012; Kypreos et al., 2014). It has been revealed that disturbance of synthesis and release of nitric oxide (eNOS is responsible for this) leads to increased aggregation of thrombocytes, inflammation of endothelium and as a result—to the disturbance of cerebral blood flow (Diodati et al., 1998; Toda et al., 2009; Austin et al., 2013). The importance of this enzyme is generally considered in the context of neurodegenerative diseases (Alzheimer's disease etc.) (Toda, 2012). However, below we will introduce recent investigations about possible role of eNOS in pathogenesis of schizophrenia.

iNOS enzyme plays an essential role in inflammatory processes during damage or infectious impairment of tissues (Lerouet et al., 2002). In the brain tissue, this type of NO synthase is located in microglia, endothelium of brain vessels, infiltrating macrophages and T-lymphocytes. Unlike eNOS and nNOS, this isoform is calcium-independent (Possel et al., 2000; Bernstein et al., 2005a). Microglia produces neuro-toxic nitric oxide (Lazzaro et al., 2014). Regulation of iNOS gene transcription depends on cAMP concentration. When level of cAMP increases, cellular reactions of inflammation are blocked (Markovic et al., 2003; Valko et al., 2007). NO, generated by inducible NOS, is involved in pathogenesis of Alzheimer's diseases, septic shock, multiple sclerosis and brain ischemia (Wass et al., 2008; Ghasemi and Fatemi, 2014).

Neuronal NOS (nNOS) generates up to 90% of nitric oxide in the brain of mammals (Hara et al., 1996). Besides neurons, nNOS is found in skeletal muscles, myocardium and smooth muscle tissue (McConell et al., 2007; Seddon et al., 2007). This enzyme is linked with NMDA receptors by means of the specific domain. This allows for activation the NO synthesis in response to influx of calcium ions into the cell after excitement of receptors (Girouard et al., 2009). Synthesis of this enzyme in the neuron is determined by the function carried out by the cell. It has been shown that nNOS influences the development of neuronal structures (Chen et al., 2004). nNOS distribution in neurons depends on their localisation. The highest concentrations of the enzyme are found in substantia innominata, septum, cortex cerebelli, hypothalamus, subthalamus. The lowest concentrations are found in corpus callosum, thalamus, occipital cortex, dentate nucleus (Blum-Degen et al., 1999). In addition to neurons, nNOS can be present in the cells of glia and blood vessels of the brain (Kawakami et al., 1998). Sufficiently detailed review of metabolism of nNOS has been published by Zhou and Zhu (2009).

### Role of nitric oxide in CNS

NO is the second mediator of activation of NMDA receptors (subtype of glutamatergic receptors). In norm, during activation of these receptors by glutamate, calcium influx into the cell is stimulated, and NO synthase forms nitric oxide that intermediately activates guanylate cyclase and causes increased cGMP synthesis (Szabadits et al., 2011). It has been identified that the concentration of NO reflects activity of glutamatergic neurotransmission (Akyol et al., 2004). However, other receptors of glutamate (for example, AMPA) also are able to generate NO (Zhou et al., 2006). This pathway modulates the release of glutamate and dopamine (Hoque et al., 2010; Szabadits et al., 2011). In addition to glutamate, NO is involved in storage, uptake and release of mediators, such as acetylcholine, noradrenaline, GABA, taurine and a glycine (Kraus and Prast, 2001; Boehning and Snyder, 2003). In addition, nitric oxide is a mediator able to activate its own extrasynaptic receptors, located some distance from the place of NO synthesis (Kiss and Vizi, 2001). It is known that nitric oxide is involved in the process of development of the nervous system (Lonart et al., 1992; Gibbs, 2003; Bernstein et al., 2011). Recent experimental studies have increased the evidence base of this statement. So, it has been established that nNOS-containing neurons actively participate in the rostral path of migration of neuroblasts, creating new synaptic connections and considerably influencing the neurogenesis (Blasko et al., 2013). Migration of astrocytes is also determined by release of NO under action of iNOS, according to an investigation by Wang et al. (2011). Nitric oxide is recognized also as an important factor of the formation of synapses and the growth of nervous fibers (Duan et al., 2012; Cooke et al., 2013; Upreti et al., 2013). It is worth repeating that NO influences the development of nervous tissue by regulation of cerebral blood flow (Furchgott and Zawadzki, 1980; Endres et al., 2004; Virarkar et al., 2013).

### Role of nitric oxide and related molecules in schizophrenia development

As has been mentioned above, nitric oxide can also act as a free radical. To this end, it is worth considering the participation of NO in the pathogenesis of schizophrenia.

For the first time, Russian scientists Averbukh et al. (1966) and Bul'ba et al. (1968) have suspected that there is an association between NO and the onset of schizophrenia (Averbukh et al., 1966; Bul'ba et al., 1968). However, this molecule has been most actively investigated in the aspect of mental diseases during the last 25 years. Identification of activity of nitric oxide in tissues and biological media can be carried out by means of detection of NO-synthase, because the NO molecule has a very short period of existence (Babicová et al., 2013).

#### Post-mortem studies

Researchers who have found an increased level of nitric oxide in post-mortem brain tissue (Yao et al., 2004; Lauer et al., 2005; Cui et al., 2010) and in plasma (Zhang et al., 2010; Flatow et al., 2013) of schizophrenics also support the existence of an association between NO synthase activity and schizophrenia. Actually, the level of nNOS differs in patients with schizophrenia and healthy controls (Akyol et al., 2004). However, this issue is disputed. In the cortex of the cerebellum, the level of nNOS did not differ between patients with schizophrenia and healthy controls in the investigation by Doyle and Slater (1995). However, there is also other data—increased NO synthase activity has been detected in Purkinje cells and dentate nucleus of patients with schizophrenia but not with depression (Bernstein et al., 2001). With regard to the neocortex, data about presence of NO synthase are inconsistent. There is data about increasing of expression of nNOS in prefrontal cortex in schizophrenia (Baba et al., 2004), but a disproof of these findings was also published (Xing et al., 2002). Interesting data were obtained during investigations of neurons of hypothalamus. So, the decreasing of nNOS-containing neurons has been revealed in periventricular nucleus of patients with schizophrenia and affective disorders (Bernstein et al., 1998). Decreased number of neurons with nNOS in suprachiasmatic nucleus has been also described (Bernstein et al., 2005b, 2010). At the same time, the expression level of enzyme was not disturbed (Bernstein et al., 2000). It is known that NO in the hypothalamus regulates synthesis and release of hormones which regulate hypothalamic-pituitary-adrenal system (HPAS): oxytocin, vasopressin, corticoliberin (Bernstein et al., 1998). Disturbed production and release of these peptides leads to HPAS hyperactivation in patients with schizophrenia (Ryan et al., 2004). The findings indicate that the disturbed NO synthesis by means of influence on hormonal balance can lead to the development of mental diseases. This statement is also confirmed by the fact that improvement of the course of depression and other mental diseases after electroconvulsive therapy is conditioned by the effect of hypothalamic NO. Rosen et al. (2003) have shown that nitric oxide during electroconvulsive therapy exerts an essential influence on cerebral blood flow, long-term potentiation of NMDA receptors, and activity of HPAS (Rosen et al., 2003). In addition to hypothalamic structures, there are data about the change of NO activity in striopallidar system (Lauer et al., 2005), nucleus caudatus, and hippocampus in mental disorders. In one instance, based on study of 15 postmortem samples of the brain of patients with schizophrenia, the decrease in the number of nNOS-containing neurons in lateral part of the lenticular nucleus has been found (Fritzen et al., 2007). The increased activity of nitric oxide in neurons of nucleus caudatus has been also shown for patients with schizophrenia (Yao et al., 2004). Research of the hippocampus indicated differences between healthy controls from schizophrenics. It was revealed that NO-synthase been activated mainly in the right hippocampus of schizophrenics (Kristofiková et al., 2008). Although currently there are less post-mortem studies, experimental studies on modeling of schizophrenia confirm increase in the concentration of NO-synthase and activation of microglial inflammation in hypothalamus (Jing et al., 2013; Ribeiro et al., 2013). Researchers who used phencyclidine for modeling of symptoms of schizophrenia in animals confirm the alteration of metabolism of L-arginine and as a result—high concentration of NO in brain structures. An association between altered content of nitric oxide and disturbances of behavior and cognition was found (Pålsson et al., 2010; Finnerty et al., 2013; Knox et al., 2014).

#### NO levels in biological fluids

Measurement of NO level in biological fluids of schizophrenics has also been carried out. Level of NOS and NO metabolites in the blood of patients with schizophrenia and depression has been found by various investigators, both as increased (Yilmaz et al., 2007; Akpinar et al., 2013) and as decreased (Srivastava et al., 2001). In the meta-analysis by Maia-de-Oliveira et al. (2012) it has been shown that the level of nitric oxide does not differ significantly between patients with schizophrenia and healthy controls. However, a reliably higher level of NO was found in patients treated with antipsychotics, which suggests the influence of these drugs on the metabolism of NO synthase (Maia-de-Oliveira et al., 2012). In addition to blood, attention of the investigators was drawn by cerebrospinal fluid. But articles on this topic are scarce, and results are not consistent. According to the investigation by Ramirez et al. (2004), in cerebrospinal fluid of patients with schizophrenia, the level of nitric oxide is decreased (Ramirez et al., 2004). Concentration of substances in cerebrospinal fluid reflects their synthesis by brain tissues. It is therefore possible to assume a decrease in NO synthesis by cells of the brain in schizophrenia. However, at this stage it is not yet possible to confirm this hypothesis. The evidence base is too small. The other known factors influencing the level of nitric oxide in tissues and fluids of the organism are as follows: smoking (Vleeming et al., 2002) and treatment by antipsychotics (Ramirez et al., 2004).

#### NO and neurodevelopment

In view of the possible association of schizophrenia and metabolism of nitric oxide in the nervous tissue, the process of neurodevelopment is of interest. There is an opinion that disturbances of the defined localisation of NOS-containing neurons in the process of development of the brain are associated with vulnerability to schizophrenia (Eastwood and Harrison, 2003). Nitric oxide was detected in some neurons from the moment of their formation, even before migration to the point of final differentiation. From the very beginning, NO plays a key role in formation of synapses and establishment of local neurotransmitter links (Gibbs, 2003). Thus, movement of such neurons to an inappropriate place leads to multiple morphological and neurochemical alterations, which is typical of schizophrenia (Lewis et al., 2005; Connor et al., 2011). In addition, the influence of overproduction of nitric oxide in prenatal and perinatal periods is also considered. It has been shown (but only in single study) that viral infection can cause abundant formation of nitric oxide in rat's brain (Fatemi et al., 2000). The increased NO synthesis in white matter leads to damage of oligodendrocytes, demyelination of fibers (this was observed in schizophrenics) (Uranova et al., 2004). Increased activity of nNOS was also described at the postnatal stage. It also leads to specific disturbances. There was an interrelationship between NO production and NMDA receptors, which are known as low-activity in patients with schizophrenia (Northoff et al., 2005). The increased formation of NO, however, does not seem to be caused by NMDA receptors. Involvement of AMPAR receptors has been considered as the most probable, especially because their high expression in schizophrenics has been found (Dracheva et al., 2005; Tanda et al., 2009). Continuous stimulation of the release of nitric oxide results in disturbed synaptogenesis, synaptic remodeling, and alterations of synaptic membranes (Sunico et al., 2005). One should not underestimate the role of nitric oxide as a free radical. Continuous stimulation of NO synthesis results in damage of membranes of cells and mitochondrions, which has been found in schizophrenia (Ben-Shachar and Laifenfeld, 2004). However, the role of overproduction of nitric oxide in development of mental does not have a sufficient evidence base as it does in neurologic pathology (multiple sclerosis, Alzheimer's disease) and should be confirmed by further research.

The decreased synthesis of nitric oxide in the brain is also discussed as a pathogenic factor of mental disorders. It has been shown that nNOS deficiency in experiments results in cognitive and behavioral disturbances, pathognomonic for schizophrenia (Kirchner et al., 2004; Dec et al., 2014). Therefore, even the migration of neurons described above may result in NO deficit in some areas and in cognitive deficiency.

The described disturbances of synthesis and release of nitric oxide were actively studied in the aspect of the hypothesis according to which schizophrenia is conditioned by disturbed neurogenesis. A paper by Oldreive et al. (2012) has shown that an increased level of nNOS and nitric oxide deteriorates the survival rate of Purkinje cells of the cerebellum *in vitro* (Oldreive et al., 2012). We have previously mentioned that the increased activity of nNOS was noticed in Purkinje cells in patients with schizophrenia (Bernstein et al., 2001). It is interesting that the harmful influence of nitric oxide on cerebellar neurons depends on stage of their development: maturation of cells makes them less sensitive to NO (Oldreive et al., 2008). The influence of nitric oxide on the development of the hippocampus is in detail discussed in recently published thematic reviews (Gray and Cheung, 2014; Hu and Zhu, 2014). As additional evidence of the participation of nitric oxide in the process of neurodevelopment, experiments serve with axotomy of motoneurons. After axotomy, the neuron starts the reparation program that includes a dephenotyping, creation of new synaptic afferent and efferent links. One of the key factors in this process is NO (González-Forero and Moreno-López, 2014). Studies have revealed that nitric oxide can induce a proliferation of neuronal stem cells *in vitro*, and at early stages—through system of ERK/MAP-kinase, and at late stages—through guanylyl cyclase-cyclic GMP-protein kinase G (Carreira et al., 2013). Research in this direction continues and promises to shed light on features of neurogenesis and its key participants.

Nitric oxide also participates in the interrelationships between neurons and gliacytes. It was shown that increased synthesis of NO by microglia can lead to damage of neurons (Graber et al., 2012). iNOS is responsible for synthesis of nitric oxide in microglia (Contestabile et al., 2012). *In vitro* studies indicated NO-dependent microglial reactions in the form of death of neurons, especially under conditions of inflammation (Graber et al., 2012). Nevertheless, neurons are able to secrete substances causing apoptosis of glia for prevention of excessive synthesis of NO (Polazzi and Contestabile, 2006). Against the background of introduced results, conclusions that iNOS activates neurogenesis in dentate gyrus of hippocampus are interesting (Luo et al., 2007). These data contradict to findings of *in vitro* research, but at the same time supplement evidence of the participation of NO-synthase in processes of neurogenesis. There are still not enough findings about the interrelationships between the activity of iNOS and schizophrenia development. However, this enzyme is important for understanding the role of microglia and inflammation in disturbances of development of the central nervous system.

## Genetic aspects of metabolism of NO within schizophrenia (Table 1)

As of right now, there are associative investigations of polymorphisms of gene of neuronal NO-synthase carried out in patients with schizophrenia (is coded by NOS1 gene). In one of the earliest papers (Shinkai et al., 2002), a reliable association between the disease and the C276T polymorphism carriage was revealed (*p* = 0.000007) (Shinkai et al., 2002). Further research included several other points: Reif et al. (2006) have established a reliable association of regulatory exon 1c promoter polymorphism as well as nNOS-mini haplotype with the development of schizophrenia and prefrontal functioning (Reif et al., 2006). One of the papers by Chinese scientists where 11 polymorphisms of *NOS1* gene are considered in 1705 patients has confirmed an association of polymorphism of rs3782206 and some haplotypes from 5′ flank region of the gene with schizophrenia (Tang et al., 2008). It is worth mentioning that there are two papers with negative results. Okumura et al. (2009) have carried out the analysis of seven polymorphisms of *NOS1* gene (rs41279104, rs3782221, rs3782219, rs561712, rs3782206, rs2682826, and rs6490121), but found that reliable associations have been recognized as type I mistake in multiple calculations, and are consequently untenable (Okumura et al., 2009). A similar situation is described for polymorphism rs1520811 in the Chinese population (Wang et al., 2012). Earlier Liou et al. (2003) have also published data on the absence of an association between CA repeat polymorphism and schizophrenia (Liou et al., 2003). Positive results for the Japanese population have been presented in analysis by Cui et al. (2010), who have revealed an association of polymorphism rs41279104 with schizophrenia development. Further, it is established that carriers of allele A have reliably earlier age of manifestation of the disease as compared with GG-homozygotes. In women, the influence of genotype on tendency to disease was expressed more strongly (Cui et al., 2010). Polymorphism rs6490121 has shown a possible association with schizophrenia onset in GWAS, which was confirmed by Riley et al. (2010). Functional polymorphisms “NOS1_1d” and “NOS1_1f” have also been studied, but on small samples of assays of brain tissue, having shown a reliable increase in expression in schizophrenia (Silberberg et al., 2010). *NOS1* gene polymorphisms were combined with disturbance of cognitive functions both in patients with schizophrenia (Donohoe et al., 2009; Reif et al., 2011) and in healthy persons (Donohoe et al., 2009; O'Donoghue et al., 2012; Rose et al., 2012). Moreover, an essential association between the carriage of G-allele of rs6490121 and reduction of thickness of gray matter of the ventromedial prefrontal cortex has been noted (Rose et al., 2012). Three investigations studied the association between carboxyl-terminal PDZ ligand of neuronal nitric oxide synthase (CAPON) gene and the onset of schizophrenia across different populations. The role of this gene consists in a disturbance of functioning of NMDA-receptors (hypofunction) that keeps within one of the theories of development of schizophrenia. Two investigations have confirmed the existence of reliable linkage disequilibrium between schizophrenia and polymorphisms of this gene (Brzustowicz et al., 2004; Xu et al., 2005). However, the British scientists could not replicate previous results (Puri et al., 2006). For association of polymorphisms of gene encoding carboxyl-terminal PDZ ligand of neuronal nitric oxide synthase (*NOS1AP*) with schizophrenia, few investigations have been carried out. The assumption of the possible role in disease development (Wratten et al., 2009; Delorme et al., 2010) was the only produced result. In summary, it is worth mentioning the meta-analysis carried out by Weber et al. (2014). The authors studied the role of polymorphisms of genes *NOS1* and *NOS1AP* in schizophrenia development. Polymorphism (SNP) rs41279104 is reliably associated with disease. The carriage of the defective allele leads to a decrease of nNOS in prefrontal cortex. The bioinformatic analysis has also confirmed an interaction between *NOS1* and *NOS1AP* in increase of the tendency to the disease (Weber et al., 2014).

**Table 1:** **Genetic studies of nitric oxide-related enzymes' in schizophrenia**.

**Gene**	**Investigated polymorphism(s)**	**Sample**	**Control**	**Ethnicity**	**Results**	**References**
*NOS1*	C276T in exon 29	215	182	Japanese	Allele frequencies of C276T differed significantly between healthy control and schizophrenics (*p* = 0.000007)	Shinkai et al., 2002
	VNTR, G-84A, rs2293054, rs1047735, rs2133681, rs2293044	195	286	German	G-84A polymorphism was linked to schizophrenia. Haplotype VNTR(N)-G84A(A) was associated with disease (*p* = 0.002)	Reif et al., 2006
	rs3782206	1705	None	Chinese	This polymorphism and several haplotypes derived from it has been associated with schizophrenia, aspeccially paranoid subgroup	Tang et al., 2008
	rs41279104, rs3782221, rs3782219, rs561712, rs3782206, rs2682826, and rs6490121	542	519	Japanese	Two SNPs were associated with schizophrenia: rs3782219 (*p* allele = 0.0291) and rs3782206 (*p* allele = 0.0124, *p* genotype = 0.0490). Authors concluded that association was the result of type 1 error of multiple testing (rs3782219: *p* allele = 0.133 and rs3782206: *p* allele = 0.168)	Okumura et al., 2009
		1154	1260		Two SNPs were associated with schizophrenia rs3782219 (*p* allele = 0.0197) and rs3782206 (*p* allele = 0.0480). Authors concluded that association was the result of type 1 error of multiple testing (rs3782219: *p* allele = 0.133 and rs3782206: *p* allele = 0.168)	
	rs1520811	382	448	Chinese	Results did not support a significant association between NOS1 gene polymorphism and schizophrenia	Wang et al., 2012
	CA dinucleotide repeat polymorphism in the 3V-UTR exon 29	198	274	Chinese	The frequencies for the NOS1 genotype (*P* = 0.372) and allele (*P* = 0.287) did not differ significantly comparing schizophrenic patients and controls.	Liou et al., 2003
	rs41279104	720	None	Japanese	Significant association between this SNP and schizophrenia was found (genotypic *p* = 0.0013 and allelic *p* = 0.0011). Additionally, the average of onset age in schizophrenic patients with the A-allele was significantly earlier than GG homozygotes (*p* = 0.018). This significance was more significant for female.	Cui et al., 2010
		26	29	Not reported	There were no significant allelic or genotypic differences among clinical groups	Silberberg et al., 2010
		43	44	German	The NOS1 schizophrenia risk genotype rs41279104 AA/AG was associated with poor cognition in patients	Reif et al., 2011
	rs6490121	1021	626	Irish	Authors detect no evidence of association with rs6490121 in NOS1 (one-tailed *p* = 0.21)	Riley et al., 2010
		349	230	Irish	GG genotype carriers performing below IQ level compared to other genotype groups	Donohoe et al., 2009
		232	1344	German		
		None	54	Irish	Carriers of the previously identified risk “G” allele showed significantly lower P1 responses (EEG-study) than non-carriers	O'Donoghue et al., 2012
		None	157	Irish	An a priori region-of-interest analysis identified a significant reduction in ventromedial prefrontal GM volume in “G” allele carriers. Risk carriers also exhibited altered patterns of activation in the prefrontal cortex, caudate, and superior parietal lobe	Rose et al., 2012
*CAPON*	*CAPON* region (15 SNPs)	85	232	Celtic and German	6 SNPs exhibited significant LD to schizophrenia (nominal *p* < 0.05).	Brzustowicz et al., 2004
	rs1415263, rs4145621, rs2661818	35	35	Not reported	For each SNP, individuals with one or two copies of the previously identified associated allele were observed to have higher group mean CAPON short-form expression than the group of individuals homozygous for the unassociated allele	Xu et al., 2005
	CAPON region (8 SNPs)	450	450	UK-based (English, Irish, Scottish, Welsh)	No evidence for allelic or haplotypic association with schizophrenia for any of the markers was found	Puri et al., 2006
*NOS1AP*	60 SNPs	85	232	Celtic and German	The rs12742393 was associated with schizophrenia. That acts by enhancing transcription factor binding and increasing gene expression	Wratten et al., 2009
	10 SNPs	72	93	French	Two non-synonymous variations, V37I and D423N were identified in two families. These rare variations apparently segregate with the presence of psychiatric conditions.	Delorme et al., 2010

## Nitric oxide as potential target for development of schizophrenia treatment

### Influence of antipsychotics on metabolism of NO

It has been shown that antipsychotics alter the metabolism of NO in the brain. Haloperidol is able to suppress activity of nNOS (Nel and Harvey, 2003; Zhang et al., 2012). However, long-term administration of drugs results in hyperactivity of this enzyme in striatum of rats (Lau et al., 2003). Authors of this study have assumed that late alterations of nNOS activity in neostriatum during treatment with an antipsychotic participate in pathogenesis of late dyskinesia (tardive dyskinesia) (Lau et al., 2003). It is worth repeating that in plasma of schizophrenics receiving antipsychotics, the activity of nNOS was higher than in healthy controls (Maia-de-Oliveira et al., 2012). These arguments call into question the existence of the uniform concept of influence of antipsychotics on the activity of nitric oxide. It is noted that atypical antipsychotics practically do not influence the activity of nNOS in brain structures of patients with schizophrenia (Tarazi et al., 2002). Besides the neuronal isoform, antipsychotics were studied in terms of influence on other NOS isoforms. Clozapine is able to inhibit activity of iNOS and to decrease the level of nitric oxide in the brain and microglial inflammation (Bringas et al., 2012; Ribeiro et al., 2013). The influence of antipsychotics on metabolism of nitric oxide leads to restoration of the normal function of NMDA-receptors. However, it is not the basic link of the mechanism of action of the medication.

### Metabolism of nitric oxide as a target for therapy of schizophrenia

Because the considerable evidence base showing that alterations of activity of NO are active in pathogenesis of schizophrenia, recent attempts have been undertaken to develop the therapy correcting disturbances of the synthesis and release of nitric oxide. Minocycline, semisynthetic tetracycline of the second generation, inhibiting enzyme iNOS and preventing development of microglial inflammatory process has been quite well studied. In double blind placebo-controlled investigations, minocycline has shown efficiency regarding negative symptoms of schizophrenia when added to antipsychotic therapy (Ghanizadeh et al., 2014; Khodaie-Ardakani et al., 2014; Liu et al., 2014). Reports about severe side effects in the carried-out protocols have not been registered. Besides, the meta-analysis of four randomized controlled investigations was published. In the meta-analysis, it has been confirmed that the use of minocycline is essentially more effective than placebo and reduces the severity of negative symptoms, but does not influence the change of values of scales of positive symptoms of PANSS (Oya et al., 2014). It is worth considering that minocycline have been studied relatively recently, and it is too early to speak to the delayed effects of medication. Therefore, it is necessary to wait for the long-term prospective investigations to conclude.

L-arginine was considered as one of the perspective targets. The carrier of L-arginine is cationic amino-acid transporter (CAT), which can also bind with L-lysine on the competitive base (White et al., 1982). Thus, L-lysine is considered as NO synthesis inhibitor in CNS. Currently, there are two publications confirming the positive role of L-lysine as an additional preparation in treatment of schizophrenia. Results of 8-week randomized double blind placebo-controlled investigation by Zeinoddini et al. (2014) have been recently published, in which Risperidone was supplemented by L-lysine. The authors noted that the medication improves indices on the following scales of PANSS: negative symptoms (*p* < 0.001), total score (*p* < 0.001), general psychopathology (*p* < 0.001). For the scale of positive symptoms, a difference was not noticed (*p* = 0.61). However, long-term effects of L-lysine were not revealed and the sample of patients in the investigation was also insignificant for final conclusions (Zeinoddini et al., 2014). Besides work by Zeinoddini et al. earlier results of a pilot investigation by Wass et al. (2011) with similar conclusions have been published (Wass et al., 2011).

## Conclusion

In the presented review of literature, recent achievements in the study of the role of nitric oxide in pathogenesis of schizophrenia and its treatment have been considered. Genetics and pharmacogenetics of NO-synthase as well as of other genes involved in the metabolism of NO are still insufficiently investigated. For achievement of results of high evidence level, carrying out research on increased samples with account for ethnic origin is necessary. Certainly, it is impossible not to take into account the exogenous factors. Possibly, their role in the development of mental disorders will remain incomplete for a long time. However, taking into account the influence of the environment should be an integral part of each investigation. The epigenetic methods, which allow for the reflection of the influence of various factors on expression of genes, look promising. Of no doubt, it is worth including epigenetic methods in complex designs for receipt of multi-sided conclusions regarding oxidative stress in pathogenesis of schizophrenia.

Disclosure of molecular-genetic mechanisms of development of schizophrenia becomes the first step in development of pathogenetic therapy. The attempts, undertaken in the field of impact on factors of oxidative stress for the purpose of improvement of the course of the disease, clearly show positive prospects of this approach. But without a sufficient fundamental basis, achievement of significant results of clinical investigations will be a failure.

### Conflict of interest statement

The authors declare that the research was conducted in the absence of any commercial or financial relationships that could be construed as a potential conflict of interest.
